# The S190R mutation in RSV-A F protein impairs nirsevimab binding and neutralization capacity

**DOI:** 10.1093/ve/veag002

**Published:** 2026-01-08

**Authors:** Sapir Cordela, Jhonatan Harari, Romila Moirangthem, Raghad Khaleafi, Orly Kladnitsky, Deborah Duran, Myriam Grunewald, Yotam Bar-On

**Affiliations:** Department of Immunology, Rappaport Faculty of Medicine, Technion-Israel Institute of Technology, Efron 1 St, Haifa 3525422, Israel; Department of Immunology, Rappaport Faculty of Medicine, Technion-Israel Institute of Technology, Efron 1 St, Haifa 3525422, Israel; Department of Immunology, Rappaport Faculty of Medicine, Technion-Israel Institute of Technology, Efron 1 St, Haifa 3525422, Israel; Department of Immunology, Rappaport Faculty of Medicine, Technion-Israel Institute of Technology, Efron 1 St, Haifa 3525422, Israel; Department of Immunology, Rappaport Faculty of Medicine, Technion-Israel Institute of Technology, Efron 1 St, Haifa 3525422, Israel; Hadassah Organoid Center, Hadassah Hebrew University Medical Center, Jerusalem 9112102, Israel; Hadassah Organoid Center, Hadassah Hebrew University Medical Center, Jerusalem 9112102, Israel; Department of Immunology, Rappaport Faculty of Medicine, Technion-Israel Institute of Technology, Efron 1 St, Haifa 3525422, Israel

**Keywords:** viral immunity, RSV variants, viral mutations, passive immunization, monoclonal antibodies

## Abstract

In 2023, the US Food and Drug Administration approved the use of nirsevimab for the prevention of respiratory syncytial virus (RSV) infections in healthy infants. This marks an important milestone for using passive immunotherapy for the prevention of viral infections. Previous studies that examined RSV-A sequences from breakthrough infection of nirsevimab-treated infants indicated that the nirsevimab binding site in the RSV-A F protein remained intact. To explore possible nirsevimab-resistant mutations that occur outside the antibody binding site, we have dissected the RSV-A mutations landscape at a single-genome resolution following exposure to nirsevimab. We identified a single amino acid substitution (S190R) at the antigenic site V of the RSV-A F protein that emerged in the vast majority of the isolated viruses. We further demonstrated that the S190R mutation reduces the binding and neutralization capacity of nirsevimab by altering antibody accessibility to site Ø epitopes. Additionally, by analysing the replication of S190R RSV-A in cell lines and in primary human organoids, we illustrated that the S190R mutation impairs the viral fitness of RSV-A. Thus, our study provides insight into possible viral mechanisms that can contribute to RSV-A escape from nirsevimab-based immunization.

## Introduction

The respiratory syncytial virus (RSV) causes a highly frequent respiratory infection, in which infants and the elderly are at higher risk of developing severe disease symptoms (Tabor et al. 2021, Baraldi et al. 2022, Domachowske 2024). Moreover, the highest rate of RSV-associated health care utilization is seen in infants during the first year of birth (Wildenbeest et al. 2023). The long-lasting attempts to develop an effective RSV vaccine have led to the US Food and Drug Administration (FDA) approval of two vaccines for the elderly population in 2023 (Walsh et al. 2023). However, as these vaccines are not approved for use in infants, their protection against RSV infection is significantly dependent on passive immunization. A stepping-stone in infant protection from RSV was recently achieved with the development of the monoclonal antibody nirsevimab (Beyfortus) for the prevention of RSV lower respiratory tract disease in all infants and in a subgroup of 2-year-old children who remain at high risk for serious disease (Fourati n.d., Ahani et al. 2023, Keam 2023, Turalde-Mapili et al. 2023, Ernst et al. 2024). Nirsevimab is a highly potent neutralizing antibody that targets the antigenic site Ø of the RSV-A F protein. Additionally, it contains three-amino acid modifications (tyrosine, threonine, and glutamate) in the Fc region that significantly extend its serum persistence and its half-life (Lee et al. 2019). This is one of the advantages of nirsevimab over the monoclonal antibody palivizumab, which has been in use for the protection of high-risk infants from RSV infection (Griffin et al. 2020).

Nirsevimab approval for use in healthy infants makes it the first monoclonal antibody to be widely used in healthy individuals as a preventative measure against a viral infection (Moirangthem and Bar-On 2025). The development of broadly neutralizing antibodies against other highly mutable viruses has made it possible to examine the potential of using passive immunization against other viral infections. This is mainly evident in the HIV-1 field, where several monoclonal antibodies are being tested in late-phase clinical trials for their ability to prevent HIV-1 infection or to control the infection (Caskey et al. 2015, Schoofs et al. 2016, Caskey et al. 2017, Escolano et al. 2017, Bar-On et al. 2018, Liu et al. 2020, Stephenson et al. 2021). Of note, the development of HIV-1-resistant strains following antibody administration has partially hindered the long-term suppression of viremia (Schoofs et al. 2016, Caskey et al. 2017). This has led to the development of antibody combination therapies that limit the escape of the virus and allow prolonged viral suppression (Bar-On et al. 2018, Mendoza et al. 2018).

Similarly, numerous studies have followed RSV infection rates in infants who were administered with nirsevimab to learn about its clinical efficacy (Fourati n.d., Griffin et al. 2020, Drysdale et al. 2023, Turalde-Mapili et al. 2023). The efficacy and safety of nirsevimab were thoroughly tested in a phase-III clinical trial in which RSV respiratory infection was monitored for 150 days after the nirsevimab injection in infants (Hammitt et al. 2022). In this trial, nirsevimab demonstrated 74.5% efficacy, with 12 infants out of 994 who were assigned to the nirsevimab group showing RSV infection that required medical attention (Hammitt et al. 2022). Later, after the 2023 FDA approval of nirsevimab, a real-world study that followed its efficacy in infants in the Yale New Haven Health System, showed 80% efficacy against RSV-related hospitalization (Xu et al. 2025). Similar nirsevimab efficacy was recorded in an additional study that followed infants who were administered with nirsevimab across several locations in Europe and in the USA (Sumsuzzman et al. 2025a).

The pressing question that will also dictate the long-term clinical success of nirsevimab is whether RSV can develop variants that are resistant to nirsevimab while maintaining their ability to circulate in the human population. This was addressed by a number of studies that have followed breakthrough infections in infants who were administered with nirsevimab (Fourati n.d., Ahani et al. 2023, Peeples and Thongpan 2023). Analysis of RSV-B sequences in these individuals revealed the emergence of RSV viruses with amino acid substitutions in the nirsevimab binding site (Fourati n.d., Ahani et al. 2023, Peeples and Thongpan 2023). However, these studies, as well as others, demonstrated that in RSV-A breakthrough infections, the nirsevimab binding site remained intact (Fourati n.d.; Ahani et al. 2023, Peeples and Thongpan 2023). This prompted us to investigate possible RSV-A F-protein mutations that are far removed from the nirsevimab binding site but that could still alter its neutralizing capacity and antiviral activity. Here, by employing a single-genome sequencing technique following RSV-A exposure to nirsevimab, we uncovered an amino acid substitution in the RSV-A F protein that can contribute to RSV-A escape from nirsevimab-based immunization.

## Materials and methods

### Cell lines and viral strains

For propagating RSV-A, we used laryngeal carcinoma HEp-2 cells (ATCC, cat #CCL-23). This cell line was also used for RSV-A infection. For measuring nirsevimab Fc effector functions, Jurkat NFAT CD16 reporter cells (InvivoGen, cat. #jktl-nfat-cd16) were used. For the generation of nirsevimab, plasmids that encode its light chain and heavy chain were used to transfect ExpiCHO-S cells (Thermo Fisher Scientific, cat. #A29133). Cell lines were grown at 37°C, 5% CO_2_ in Dulbecco's Modified Eagle's Medium (DMEM) or Roswell Park Memorial Institute (RPMI) 1640 medium supplemented with 4500 mg/L D-glucose, 4 mM L-glutamine, 110 mg/L sodium pyruvate, 10% fetal bovine serum (FBS), 1% penicillin–streptomycin, and 1% nonessential amino acids (NEAAs). For antibody production, the ExpiCHO-S cells were maintained in 8% CO_2_ at 37°C and 120 rpm using the ExpiCHO-S cell expression medium (Thermo Fisher Scientific, cat. #A2910001). The human RSV strain A2 (RSV-A), which was isolated in 1961 from the lower respiratory tract of an infant with bronchiolitis and bronchopneumonia in Melbourne, Australia, was purchased from ATCC (American Type Culture Collection, cat. #VR15–40) and was propagated in HEp-2 cells.

### Nirsevimab cloning and purification

Nirsevimab light-chain and heavy-chain variable sequences were retrieved from patent WO 2023/039584 A2. Nirsevimab heavy- and light-chain sequences were ordered as gBlocks Integrated DNA Technologies (IDT) and were cloned into mammalian expression vectors containing either mouse IgG1-heavy and mouse κ-light chains (Moirangthem et al. 2024) or human IgG1-heavy and human κ-light chains (Von Boehmer et al. 2016). The Fc region of the human IgG1 was further modified by site-directed mutagenesis to include the YTE amino acid substitutions (M252Y/S254T/T256E) (Grevys et al. 2015). All plasmids were sequenced before they were used to transfect ExpiCHO cells following the manufacturer’s Max Titer protocol. The supernatant was collected at Day 7 after transfection. Cell viability was monitored throughout the 7 days and was kept above 90%. To purify nirsevimab from the cell supernatant, we used affinity purification with protein G agarose beads (Pierce, cat #89927), and the antibody was dialysed overnight in PBSX1 and filter-sterilized (0.22 μm). Nirsevimab purity was assessed by Sodium Dodecyl Sulfate Polyacrylamide Gel Electrophoresis (SDS-PAGE) and InstantBlue Coomassie protein stain (Abcam, cat #ab119211), as well as by Enzyme-Linked Immunosorbent Assay (ELISA) against mouse or human IgG1, and by flow cytometry analysis of nirsevimab staining of uninfected and infected HEp-2 cells.

### Viral infection and neutralization assay

For viral infection, 7 × 10^4^ HEp-2 cells were seeded in a 24-well plate with DMEM containing 10% nonheat-inactivated FBS. After overnight incubation, the cell supernatant was removed and replaced with 300 μl of DMEM with 2% nonheat-inactivated FBS containing RSV-A. After 2 h incubation at 37°C, 5% CO_2_, 1 ml of DMEM with 2% nonheat-inactivated FBS was added to the well. Analysis of infection was performed by flow cytometry 72 h after the addition of the virus. To test the neutralization levels of nirsevimab, the virus was incubated with various concentrations (indicated in the figure) of nirsevimab or PBSX1 as a control for 1 h at 37°C. The virus–nirsevimab mixture was then added to HEp-2 cells as described above, and infection was analysed by flow cytometry 72 h after the addition of the virus. In all of the flow cytometry analyses, dead cells were excluded by staining with viability dye (Thermo Fisher Scientific, cat. #65-0865-14). For the calculation of nirsevimab IC_50,_ we used a nonlinear regression model with one-phase decay (GraphPad Prism v10.4.2 software). Before infection, viruses were kept at −80°C with freezing media containing 0.4 M sucrose, 7.6 mM KH_2_PO_4_, 14.4 mM K_2_HPO_4_, and 10.8 mM monosodium glutamate.

### Serial passage of RSV-A with nirsevimab

7 × 10^4^ HEp-2 cells were seeded in a 24-well plate with DMEM containing 10% nonheat-inactivated FBS. After overnight incubation at 37°C, 5% CO_2_, the cell supernatant was removed and replaced with 300 μl of DMEM with 2% nonheat-inactivated FBS containing RSV-A. Prior to the addition of the virus, RSV-A was incubated with either PBSX1 or nirsevimab at an IC_50_ concentration for 1 h at 37°C. After 2 h of incubation of the HEp-2 cells with the virus-nirsevimab mixture at 37°C, 5% CO_2_, 1 ml of DMEM with 2% nonheat-inactivated FBS was added to the well. Every 72 h, the percentage of infection was analysed by staining the infected cells with RSV3216 antibody (Abcam, cat. #ab24011), and the cell supernatant was harvested. One millilitre of the supernatant was kept at −80°C, while the rest was immediately used to infect 7 × 10^4^ HEp-2 cells that had been seeded a day earlier in a 24-well plate, after incubation with either PBSX1 or nirsevimab at IC_50_ concentration. This was repeated every 3 days for a period of 39 days since the initial infection. Frozen supernatants collected at Days 12, 21, and 39 were used for sequencing of the progeny viruses. This assay was repeated twice in independent experiments using two different viral stocks of RSV-A2.

### Flow cytometry staining of infected cells and transfected cells

Analysis of RSV-A infection was evaluated 72 h after incubation of the cells with the virus, unless indicated otherwise. HEp-2 cells, either uninfected or infected with RSV-A, were harvested, centrifuged at 1500 RPM for 5 min, and then incubated with 100 μl of Fluorescence-Activated Cell Sorting (FACS) buffer (PBSX1 with 1% bovine serum albumin) containing one of the following antibodies: RSV3216 (Abcam, cat. #ab24011), RSV133 (Abcam, cat #ab94966), or nirsevimab (0.2 mg/ml). The cells were incubated for 45 min at 4°C and were then washed with 100 μl of FACS buffer. Cells were then stained with the secondary antibody Alexa Fluor 488-conjugated donkey anti-mouse IgG (Jackson, cat. #715-545-151) and with viability dye (Thermo Fisher Scientific, cat. #65–0865-14) for 30 min at 4°C. Cells were then washed twice with 200 μl of FACS buffer and were analysed with the BD Biosciences LSRFortessa flow cytometer. Further analysis was performed using FlowJo software v10.7. For experiments in which various RSV-A F protein antibodies were analysed, the same assay was used but with the following primary antibodies: D25 (AntibodySystem, cat. #RVV02809), 101-F (ACROBiosystems cat. #RSF-M309a), 131-2A (Sigma-Aldrich, cat. #MAB8599), and palivizumab (Invitrogen, cat. #MA560177).

For expression of RSV-A F proteins, HEp-2 cells were transfected with wild-type or mutant RSV-A F proteins that were cloned into the lentiMPH v2 vector (Addgene cat. #89308). Transfections were performed in 6-well plates using 10 μg plasmid DNA per well and the Mirus transfection reagent (Mirus, cat. #MIR 2304) following the manufacturer’s protocol. Mock-transfected controls received an equivalent amount of empty lentiMPH v2 plasmid. Cells were incubated for 48 h post-transfection, after which transfection efficiency and protein expression were evaluated by flow cytometry. All other flow cytometry analyses were done by gating on the HEp-2 cell populations that were positive for the F protein. For staining with IGF1R or nucleolin (NCL), IGF1R-Ig (Acro, cat. #IGR-H5253) and nucleolin-Ig (Acro, cat. #NUL-H5253) were used.

### Single-genome sequencing of the RSV-A F gene

Single-genome sequencing was performed as we previously described for the influenza virus and HIV-1 (Horwitz et al. 2017, Bar-On et al. 2018, Moirangthem et al. 2024). Viral RNA was isolated from the frozen supernatants collected at Days 12, 21, and 39 using a QIAamp Viral RNA Mini Kit (Qiagen, cat. #52906). complementary DNA (cDNA) was synthesized using the FW-Out primer and SuperScript III Reverse Transcriptase (Invitrogen, cat. #18080–044). The reaction was performed at 55°C for 1 h, terminated by heat inactivation at 70°C for 15 min, and finally treated with RNaseH (Invitrogen, cat. #EN0201) for 20 min at 37°C.

The cDNA was serially diluted and subjected to two rounds of nested Polymerase Chain Reaction (PCR) using the Out- and In-primer sets of the F gene. The first round of PCR amplification was performed using the 5′-Out-forward (ATGGAGTTGCTAATCCTCAA) and 3′-Out-reverse (ATGCAATATTATTTATACCACTCAG) primers. The PCR product from the first round was used as a template for the second round of PCR amplification using the In-primer set, 5′-In-forward (ACTGCAGTCACATTTTGTTT) and 3′-In-reverse GTGACTGGTGTGCTTCTG. The F gene was amplified using DreamTaq (Thermo Fisher Scientific, cat. #K1082) and analysed for the correct size of ~ 1700 bp using gel electrophoresis to confirm the right amplicon. To ensure single-genome amplification, the cDNA was serially diluted so that no more than 30% of wells were considered for library generation (according to the Poisson distribution) (Salazar-Gonzalez et al. 2008).

The amplified single genomes were used to prepare libraries by Illumina Nextera DNA Sample Preparation Kit. Sequencing was performed using the Illumina MiSeq Nano 300 cycle kits, and gene alignments were generated using Geneious 9.1.8 (Biomatters). The RSV-A F gene (UniProt P03420) was used as a reference genome. Pie charts of the mutation analysis were generated in GraphPad Prism v10.4.2 software, and Logo plots were generated using the longitudinal antigenic sequences and sites from the intrahost evolution (LASSIE) tool (Hraber et al. 2015).

### Jurkat NFAT CD16 reporter assay

CD16 (FcγRIIIa) engagement by the various antibodies was evaluated by using the Jurkat NFAT CD16 reporter cells (InvivoGen, cat. #jktl-nfat-cd16) as previously described (Moirangthem et al. 2024). After CD16 expression was confirmed by FACS staining using anti-CD16 antibody (BioLegend, cat. #980104), uninfected and RSV-A-infected HEp-2 cells were incubated with nirsevimab for 1 h at 37°C, 5% CO_2_. Addition of nirsevimab was done 72 h after infection, and 7.5 μg/ml nirsevimab with mutated human Fc was used (YTE mutations). The cells were washed and incubated with 2 × 10^5^ Jurkat NFAT CD16 reporter cells for 6 h at 37°C, 5% CO_2_. Next, 25 μl of supernatant was drawn from each well and transferred to an opaque, black, 96-well plate to which 50 μl of QuantiLuc substrate was added, and luminescence was immediately read on a microplate reader (Infinite M200 PRO). Uninfected HEp-2 cells that were incubated with the same concentration of nirsevimab were used as a control.

### Viral RNA isolation and real-time PCR

Two hundred microlitres of supernatant of HEp-2 cells was collected 72 h after infection with viruses from passages 0, 1, 2, and 3, and viral RNA was extracted using a QIAamp RNA Extraction Kit (Qiagen). Supernatant from uninfected cells was used as a control. An equal volume of extracted RNA (16 μl) was then used to generate cDNA using a LunaScript RT SuperMix Kit (NEB cat. #E3010). cDNA was then used for Reverse Transcription quantitative Polymerase Chain Reaction (RT-qPCR) using PowerTrack SYBR Green Master Mix (Thermo Fisher Scientific, cat. #A46109). For the quantification of the RSV-A L gene (RNA polymerase), the primer set used was forward (GTGCTGGATGAACTGCATGG) and reverse (GTCCACAGTTTTTGACACCACC). One microlitre of cDNA diluted 1:10 was used for each reaction. quantitative Polymerase Chain Reaction (qPCR) was done using the Bio-Rad CFX Connect Real-Time System machine with the following cycling conditions: 95°C for 2 min and 40 cycles of 95°C for 15 s and 60°C for 1 min. Results were analysed using the Bio-Rad CFX Manager 3.1 software. The same method was used for viral load measurements in mice lungs. Six-week-old C57BL/6 mice were infected intranasally with 10^8^ RSV genome copies per mouse. Lung mice were harvested at Day 5. All animal experiments were approved by the Technion Animal Care and Use Committee (#IL1100725H).

RSV-A genome copies in viral stocks were quantified by RT-qPCR. Viral RNA was extracted as described above. Quantification was performed using primers and a probe targeting the RSV-A L gene (described above). A genomic RSV-A RNA standard (ATCC-VR-1540DQ) was used to generate a 10-fold serial dilution standard curve for absolute quantification. Genome copies per reaction were converted to genome copies per millilitre based on the extraction and reaction volumes. All samples were run in technical triplicates, and standard curves consistently showed amplification efficiencies of over 90%.

### Modelling of RSV-A F protein

Three-dimensional structural models were analysed using the Mol* Viewer software (Sehnal et al. 2021) to visualize the interaction of nirsevimab with the RSV-A F protein, as well as the conformational states of the F protein alone. The structure of the nirsevimab-RSV F complex was retrieved from the Protein Data Bank (PDB 5UDC) and used to examine the antibody’s binding site and interaction interface. Separately, structural models of the RSV F protein in its prefusion (pre-F, PDB 4MMU) and postfusion (post-F, PDB 3RRT) conformations were also obtained from the PDB and were visualized independently. These models were used to assess the conformational differences and potential epitope accessibility relevant to nirsevimab binding and neutralization. For modelling the structure of the RSV-A F trimers, we used AlphaFold2 (v2.3) in multimer mode, generating five independent predictions without template structures (Hegedűs et al. 2022).

### Human nasal organoids

Nasal inferior turbinate brushings were obtained from a healthy volunteer, in accordance with protocol #HMO-0921-20 approved by the Hadassah Medical Organization Ethics Committee and the Israeli Ministry of Health. The samples were maintained in AdDF^+++,^ which is Advanced DMEM/F12 (Thermo Fisher Scientific, cat. #12634010) supplemented with 1% antibiotic–antimycotic (Capicorn Scientific, cat. #AAS-B-CAPRI), N-2-hydroxyethylpiperazine-N-2-ethanesulfonic acid (HEPES) 1 mM (Thermo Fisher Scientific, cat. #15630056), 1X GlutaMAX (Thermo Fisher Scientific, cat. #35050061), and ROCK inhibitor Y-27632 10 μM (MedChemExpress, cat. #HY-10583-50MG) until processed within 4 h following a protocol adapted from Sachs et al. (Sachs et al. 2019).

Briefly, biopsies were enzymatically digested for 30–40 min at 37°C in AdDF^+++^ supplemented with 0.5–1 mg/ml Collagenase IV (Thermo Fisher Scientific, cat. #17104019), 1 U/ml Dispase (Corning, cat. #WBD-354235), and 100 μg/ml DNase I (Roche, cat. #11284932001). Digestion was stopped by adding 1:1 volume of fetal bovine serum (Sigma-Aldrich, cat. #F7524-500ML), and the cell suspension was filtered through a 100 μm cell strainer, washed in AdDF^+++,^ and centrifuged at 200 rcf for 5 min at 4°C. Red blood cells were lysed using a red blood cell lysis buffer (Roche, cat. #11814389001), and the remaining cells were washed with ice-cold AdDF^+++^.

The cell pellet was resuspended in ice-cold growth factor–reduced (GFR) Matrigel matrix (Corning cat. #FAL356230) at a concentration of 1.6 × 10^6^ cells/ml and seeded into a 24-well culture plate. After solidification, droplets were incubated at 37°C in a humidified incubator with 5% CO_2_ in the expansion medium defined as AdDF^+++^ supplemented with 10 μM ROCK inhibitor (MedChemExpress, cat. #HY-10583-50MG), 500 nM ALK inhibitor (A83-01 Sigma-Aldrich, cat. #SML0788-5MG), 1X B27 supplement (Thermo Fisher Scientific, cat. #17504044), 1.25 mM *N*-acetyl-L-cysteine (Sigma-Aldrich, cat. # A9165-5G), 5 mM nicotinamide (Sigma-Aldrich, cat. #N0636-100G), 200 ng/ml recombinant human FGF-10 (Thermo Fisher Scientific, cat. #100-26-500), 50 ng/ml FGF-7 (Thermo Fisher Scientific, cat. #100-19-100), 100 ng/ml Noggin (Thermo Fisher Scientific, cat. #120-10C-01 M), 500 ng/ml R-spondin-1 (Thermo Fisher Scientific, cat. #120-38-500UG), and 1 μM p38 MAP kinase inhibitor (SB202190, Sigma-Aldrich, cat. #S7076-5MG).

Three-dimensional organoid structures were typically formed within the first 4 days of culture in a 37°C, 5% CO₂ incubator. During expansion, the medium was replaced twice a week. Before RSV-A infection, organoids were collected and washed in ice-cold AdDF^+++^ and disrupted by digestion with TrypLE Express (Thermo Fisher Scientific, cat. #12604013). Cells were then incubated with the indicated amounts of RSV-A in a 37°C, 5% CO₂ incubator for 2 h and then further incubated with 1 ml of DMEM with 2% nonheat-inactivated FBS in a 24-well plate for 72 h. Three uninfected organoids and three organoids infected with either Wild Type (WT) RSV-A or S190R RSV-A were used, and each group was pooled together for flow cytometry analysis.

### Statistical analysis

Statistical analyses were performed using Student’s *t*-test or one-way Analysis of Variance (ANOVA) for multiple comparisons. The method used is depicted in the figure legend, along with details on the number of biological replicates. Corrections for multiple comparisons are also specified for relevant experiments. Results are reported as mean ± SEM, unless indicated otherwise. Data analysis was conducted using GraphPad Prism v10.4.2 software.ml.

## Results

### Serial passaging of RSV-A with nirsevimab

**Figure 1:**
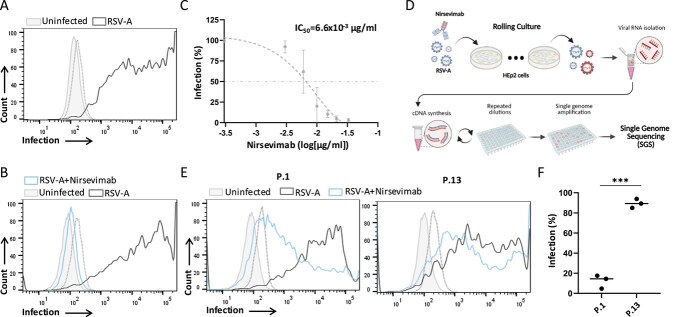
Nirsevimab and RSV-A culture system. (A) Staining of uninfected HEp-2 cells (filled histogram) and RSV-A-infected HEp-2 cells (empty histogram) with RSV3216 antibody. The dashed empty histogram depicts the staining of infected cells with secondary antibody only. Shown is one representative experiment out of five that were performed. (B) Staining of uninfected HEp-2 cells (filled histogram) and RSV-A-infected HEp-2 cells (empty histogram) with RSV3216 antibody. The histogram named RSV-A+Nirsevimab depicts the staining of RSV3216 antibody when RSV-A was incubated with 0.02 μg/ml of nirsevimab prior to the incubation with the HEp-2 cells. The dashed empty histogram depicts the staining of infected cells with secondary antibody only. Shown is one representative experiment out of three that were performed. (C) Nirsevimab IC_50_ measurements. The *x*-axis depicts the nirsevmiab concentrations that were used. each dot depicts the average of three measurements from two independent experiments with standard errors. The IC_50_ value is indicated in the figure and was calculated by a nonlinear regression model with one-phase decay (GraphPad Prism v10.4.2 software). (D) Scheme of nirsevimab-RSV-A culture system and the single-genome sequencing of RSV-A variants that were isolated from the culture. (E) Staining of uninfected HEp-2 cells (filled histogram) and RSV-A-infected HEp-2 cells (empty histogram) with RSV3216 antibody and with RSV-A from passage #1 (upper panel) and RSV-A from passage #13 (lower panel). The histogram named RSV-A+Nirsevimab depicts the staining of RSV3216 antibody when RSV-A was incubated with 6 × 10^−3^ μg/ml of nirsevimab prior to the incubation with the HEp-2 cells. The dashed empty histogram depicts the staining of infected cells with secondary antibody only. Shown is one representative experiment out of three that were performed. (F) A summary of three independent experiments of the staining shown in (E). The *y*-axis depicts the percentage of cells that were infected. The *x*-axis depicts the passage number. Statistically significant differences are shown (^***^P < .001, Student’s *t*-test).

To thoroughly analyse the dynamic interaction of RSV-A and nirsevimab, we have cloned the Fab regions of nirsevimab into plasmids encoding the light chains and heavy chains of human IgG1 and into plasmids encoding the light chains and heavy chains of mouse IgG1 (Von Boehmer et al. 2016, Moirangthem et al. 2024). Additionally, the Fc region of the human IgG1 was modified to include the YTE mutations (M252Y/S254T/T256E), which are required for maintaining nirsevimab extended half-life (Lee et al. 2019). Plasmids were then used to transfect ExpiCHO cells, and nirsevimab was purified from the cell supernatant using protein G beads (Widjojoatmodjo et al. 1993). To verify the successful generation of nirsevimab, laryngeal carcinoma HEp-2 cells were infected with RSV strain A2 (RSV-A) and were stained with nirsevimab 72 h postinfection. Significant staining of the infected-HEp-2 cells was observed with no staining of the uninfected cells (Fig. 1A). Furthermore, we have verified the neutralization capacity of nirsevimab. RSV-A was incubated with different concentrations of nirsevimab for 1 h prior to incubation with HEp-2 cells (Fig. 1B and C). Nirsevimab successfully neutralized RSV-A with a half maximal inhibitory concentration (IC_50_) of 6.6 x 10^−3^ μg/ml (Fig. 1B and C).

Next, to examine the mutation profile of the RSV-A F protein in response to nirsevimab, we passaged RSV-A in HEp-2 cells with or without an IC_50_ concentration of nirsevimab (Fig. 1D). Every 3 days, the cell supernatant was collected, and the infected cells were evaluated for the level of infection by staining the cells with RSV3216 antibody (Fig. 1D–F). By comparing the levels of HEp-2 cells infection by passage 1 and passage 13 viruses in the presence or absence of an IC_50_ concentration of nirsevimab, we observed that passage 13 viruses exhibited significantly reduced sensitivity to nirsevimab (Fig. 1E and F). Based on this, viruses were collected at Days 12, 21, and 39 of the rolling culture for further analysis. For in-depth evaluation of RSV-A F protein mutations that have emerged in response to nirsevimab, the isolated viruses were subjected to single-genome sequencing (SGS) of the F gene (Fig. 1D) as previously described (Bar-On et al. 2018, Khateeb et al. 2022, Moirangthem et al. 2024).

### Single-genome sequencing analysis of RSV-A viruses revealed the accumulation of S190R F protein mutants following exposure to nirsevimab

**Figure 2:**
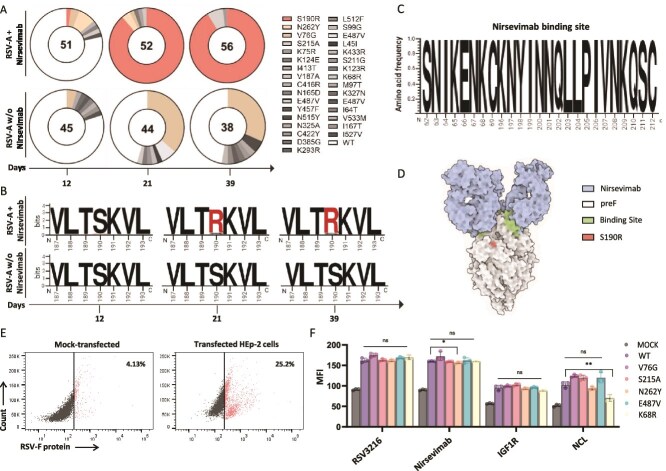
Single-genome sequencing and nirsevimab escape mutation analysis. (A) Pie charts that depict the various RSV-A variants that were recorded on Days 12, 21, and 39 of the culture. The upper panel depicts the culture of RSV-A with nirsevimab, and the lower panel depicts the culture of RSV-A without nirsevimab. The number in the middle of the pie chart depicts the total number of viruses that were analysed by SGS in each group per day. The pie slices represent the acquired mutations of the F gene as indicated in the figure. The sequences were collected from two independent rolling culture experiments using different viral stocks. (B) Logo plots that depict the various RSV-A variants that were recorded on Days 12, 21, and 39 of the culture. The upper panel depicts the culture of RSV-A with nirsevimab, and the lower panel depicts the culture of RSV-A without nirsevimab. The frequency of viruses carrying a specific mutation in the RSV F protein is shown by the height of the letter. The numbers in the *x*-axis depict the amino acid location in the RSV-A2 F protein (UniProt P03420). The figures were made using the web logo (https://weblogo.berkeley.edu/logo.cgi). The *y*-axis height specifies the number of bits, which indicates the information content of a sequence position. By default, the height of the *y*-axis is the maximum entropy for the given sequence type (log_2_ 20 = 4.3 bits for protein). The day of the analysis is also indicated on the *x*-axis. (C) Logo plot that depicts the nirsevimab binding site in the RSV-A F protein in all viruses that were analysed by SGS. The height of the letter is proportional to the frequency of each amino acid out of all viruses that were sequenced. The *x*-axis depicts the amino acid location in the RSV-A2 F protein (UniProt P03420). (D) RSV-A F pre-fusion trimer bound by nirsevimab from the PDB 5UDC structure. (E) Expression of RSV-A F protein in HEp-2 cells. Dot plots that depict the expression of RSV-A F protein (RSV3216 antibody) in mock-transfected cells and in the transfected cells 48 h after transfection. The percentage depicts the cells that were stained positively with RSV3216 antibody. (F) Staining of the mock-transfected cells and the transfected HEp-2 cells with: RSV3216, nirsevimab, IGF1R-Ig, and NCL-Ig. Statistically significant differences are shown (^*^P < .05, ^**^P < .01, ns, nonsignificant, one-way ANOVA). Shown is a summary of three independent experiments. The *y*-axis depicts the mean fluorescence index (MFI) that was recorded with each antibody.

A total of 159 viral genomes were analysed for mutations in the RSV-A F gene from the nirsevimab-treated culture, and 127 viral genomes were analysed from the untreated culture at Days 12, 21, and 39 (Fig. 2A; Supplementary Table S1). After three serial passages (Day 12) of RSV-A in untreated HEp-2 cells and in the nirsevimab-treated culture, expansion of several RSV-A variants has been recorded (Fig. 2A; Supplementary Table S1). In the untreated HEp-2 cells, we observed several RSV-A variants that emerged, but most of these variants did not further accumulate with time. However, one mutant (V76G) in the untreated culture was identified in increasing frequency from Day 12 to Day 21 and remained abundant also at Day 39 (Fig. 2A). The V76G mutation was not previously associated with any specific virological or functional phenotype. It was also identified in the nirsevimab-treated culture but did not accumulate with time and was not detected at the last day of analysis. Interestingly, in the nirsevimab-treated culture, a different mutation profile was observed in comparison with the untreated culture. The most significant difference was in variant S190R, which was completely absent in the untreated culture but was the predominant clone in the nirsevimab-treated culture (Fig. 2A and B). The S190R variant was first detected after three serial passages, has further expanded, and was the dominant clone at Day 21 with 91% of the isolated viruses carrying the S190R mutation (Fig. 2A and B). The S190R variant was also the dominant variant at a later time point of the nirsevimab-treated culture, with 93% of the isolated viruses carrying this mutation at Day 39 (Fig. 2A and B). Furthermore, our SGS analyses have indicated that the few variants that did not carry the S190R mutation had a different amino acid substitution, S215A (Fig. 2A).

In accordance with previous clinical reports that examined RSV-A sequences during breakthrough infections in nirsevimab-treated individuals (Fourati n.d., Ahani et al. 2023) the nirsevimab binding site has remained intact in all of the 286 viruses that we had isolated (Fig. 2C). Further investigation of the S190R mutation that emerged in response to nirsevimab has indicated that this mutation is located at antigenic site V of the RSV-A F protein and that it is adjacent to the binding site of nirsevimab (Fig. 2D). Thus, we concluded that the exposure of RSV-A to nirsevimab in culture has led to the emergence and the expansion of variants that carry the S190R mutation outside of nirsevimab binding site.

To gain further insights into additional amino acid substitutions that were identified in our analysis, we have cloned the RSV-A F protein of the nirsevimab-binding site variant K68R that was isolated from the untreated culture, N262Y and S215A variants that were constantly detected only in the nirsevimab-treated culture, E487V that was detected in both untreated and treated, and one variant that was found frequently in the untreated culture, V76G. The WT RSV-A F protein and these five mutated F proteins were cloned into a mammalian expression vector and were used to transfect HEp-2 cells. Expression was verified by flow cytometry 48 h after transfection (Fig. 2E). We next tested the effects of the F protein mutations on various parameters: F protein expression, nirsevimab binding, and the binding of the F protein to its known cellular receptor IGF1R and nucleolin (by staining with IGF1R-Ig and NCL-Ig) (Griffiths et al. 2020). By comparing the staining of the mock-transfected and the transfected HEp-2 cells with these antibodies, we concluded that none of these mutations significantly affected nirsevimab binding (Fig. 2F). Of note, this was also evident in the nirsevimab-binding site variant K68R, in which no significant reduction in nirsevimab binding was observed, but a significant reduction in NCL-Ig staining was recorded (Fig. 2F).

### The RSV-A F protein S190R mutation impairs nirsevimab binding, neutralization, and effector functions by masking site Ø epitopes

**Figure 3:**
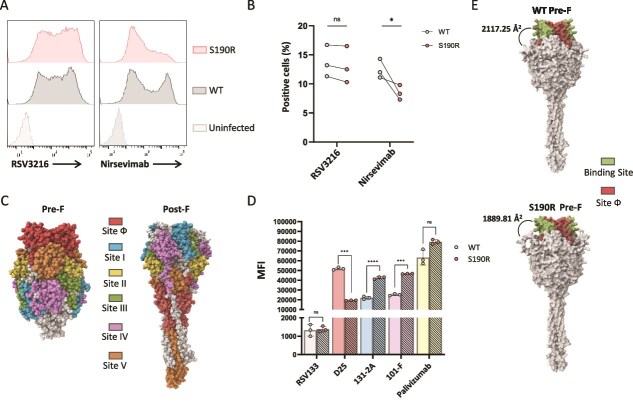
Antibody recognition of WT RSV-A F protein and S190R RSV-A F protein. (A) Staining of WT RSV-A-infected HEp2 cells and S190R RSV-A-infected HEp2 cells with RSV3216 (left panel) and with nirsevimab (right panel). Shown is one representative experiment out of three that were performed. (B) A summary of three independent experiments of the staining shown in (A). The *y*-axis depicts the percentage of cells that were stained positively with the antibodies that are indicated on the *x*-axis. Statistically significant differences are shown (^*^P < .05, ns, nonsignificant, Student’s *t*-test). (C) RSV-A F prefusion trimer (PDB 4MMU) and postfusion trimer (PDB 3RRT) illustrated by Mol^*^ Viewer software. The six different F protein antigenic sites are colour-coded and are indicated in the figure. (D) Staining of WT RSV-A-infected HEp2 cells and S190R RSV-A-infected HEp2 cells with a panel of monoclonal antibodies. The *x*-axis depicts the monoclonal antibodies that were used for staining. The *y*-axis depicts the mean fluorescent index (MFI) that was recorded with each antibody. The columns are colour-coded based on the RSV F antigenic site that is targeted by the antibody. The figure shows a summary of three independent experiments, each depicted by a dot with standard errors. Statistically significant differences are shown (^***^P < .005, ^****^P < .001, ns, nonsignificant, Student’s *t*-test). (E) Structure of the WT RSV-A F trimer and S190R F trimer as predicted by AlphaFold2 (v2.3) and analysed by Mol^*^ Viewer software. The average accessible surface area of nirsevimab binding site is indicated in the figure.

In order to test the effects of the S190R mutation on the antiviral activity of nirsevimab, we first isolated the S190R RSV-A variant from Day 39 culture (Fig. 2A). Next, we infected HEp-2 cells with various genome copies of WT RSV-A and S190R RSV-A and evaluated the level of infection by staining the cells with a monoclonal antibody, RSV133, which recognizes the RSV-G protein. We observed that infection of HEp-2 cells with 10^7^ genome copies of WT RSV-A and 2 × 10^7^ genome copies of S190R RSV-A resulted in similar infection, as indicated by the staining of the RSV-G protein on the surface of the infected-HEp-2 cells (Fig. S1). Additionally, the S190R mutation did not alter the binding of the monoclonal antibody RSV3216, which recognizes the RSV-A F protein (Fig. S1). However, despite similar infection, a significantly reduced binding of nirsevimab was seen in HEp-2 cells infected with S190R RSV-A in comparison with HEp-2 cells infected with WT RSV-A (Fig. 3A and B).

Since the S190R mutation is located outside of the nirsevimab binding site, we postulated that the reduced binding of nirsevimab is due to structural changes in the RSV-A F protein trimer. The RSV-A F trimer is composed of six well-characterized antigenic domains (Fig. 3C) (Tang et al. 2019), four of which are recognized by different monoclonal antibodies: site Ø is recognized by D25, site I is recognized by 131-2A, site II is recognized by palivizumab, and site IV is recognized by 101-F (Widjaja et al. 2016). We used an array of these antibodies to stain HEp-2 cells infected either with WT RSV-A or with S190R RSV-A, after verifying similar infection levels by staining with RSV133 (Fig. 3D). No significant changes were observed in palivizumab (site II) binding to S190R RSV-A-infected cells, and the binding of 131-2A (site I) and 101-F (site IV) was even increased in S190R RSV-A-infected cells compared to WT RSV-A-infected cells. However, the binding of D25, which, similarly to nirsevimab, targets epitopes in site Ø, was significantly reduced in S190R RSV-A-infected cells compared with WT RSV-A-infected cells (Fig. 3D). Thus, the accessibility of antibodies to site Ø is impaired in the S190R RSV-A F trimer. Consistent with this, structural modelling using AlphaFold2 (v2.3) and Mol^*^ Viewer software (Hegedűs et al. 2022) further demonstrated that the S190R substitution decreases the average accessible surface area of the nirsevimab binding site (WT = 2117.25 Å^2^; S190R mutant = 1889.81 Å^2^) (Fig. 3E).

**Figure 4:**
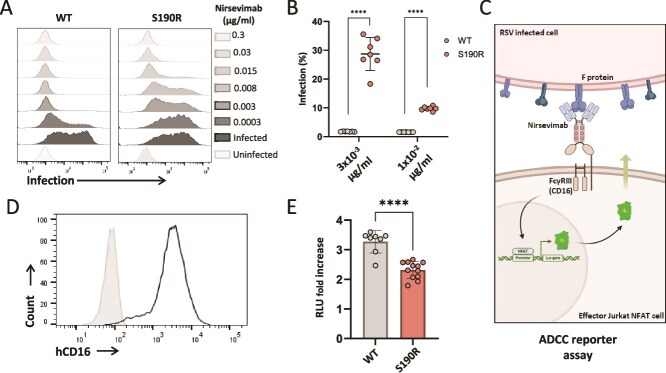
Testing nirsevimab antiviral activity against WT RSV-A and S190R RSV-A. (A) Staining for RSV-A-infected cells (RSV3216 antibody) in the absence or presence of nirsevimab at different amounts that are depicted in the figure. The left panels show the level of infection of WT RSV-A, and the right panels show the level of infection with S190R RSV-A. (B) Nirsevimab neutralization against WT and S190R RSV-A. The *x*-axis depicts the concentrations of nirsevimab that were used for neutralization. Each dot depicts an independent experiment. Statistically significant differences are shown (^****^P < .001, Student’s *t*-test). (C) Scheme of the Jurkat NFAT hCD16 ADCC reporter assay. (D) Staining of Jurkat NFAT hCD16 cells with anti-human CD16 antibody. The grey histogram depicts the background staining of Jurkat NFAT hCD16 cells, and the empty black histogram depicts the staining with anti-human CD16 antibody. (E) Luminescence values (relative luminescence units, RLUs) following incubation of Jurkat NFAT CD16 cells with uninfected HEp-2 cells, WT RSV-A-infected HEp-2 cells, and S190R RSV-A-infected cells and after incubation of the cells with nirsevimab. Shown are fold changes of the luminescence values seen for the infected cells. Uninfected HEp-2 cells were used as control and were set as 1. Shown is a summary of three independent experiments out of three performed. Each dot depicts a tested well, and error bars are shown. Data represent mean ± SEM. Statistically significant differences are shown (^****^P < .0001, Student’s *t*-test).

The reduced binding of nirsevimab observed in S190R RSV-A-infected cells has prompted us to test if this RSV-A F protein mutation also alters nirsevimab neutralization capacity and effector functions. WT RSV-A (10^7^ genome copies) and S190R RSV-A (2 × 10^7^ genome copies) were incubated with varying amounts of nirsevimab ranging from 0.3 to 300 ng per well and were then used to infect HEp-2 cells. In the absence of nirsevimab, no significant changes were seen between infection with WT RSV-A or with S190R RSV-A (Fig. 4A). Nevertheless, nirsevimab demonstrated a significantly lower neutralization of the S190R RSV-A compared to WT RSV-A, across various nirsevimab concentrations (Fig. 4A and B).

Next, in order to compare the effector functions of nirsevimab toward HEp-2 cells infected with either WT RSV-A or S190R RSV-A, we have used Jurkat NFAT CD16 reporter cells stably expressing the human FcγRIIIa (hCD16) (Moirangthem et al. 2024). These cells are engineered to have an NFAT-inducible Lucia luciferase reporter gene that, upon human CD16 activation by the antibody Fc domain, mediates NFAT activation (Fig. 4C). After verification of surface human CD16 expression on the Jurkat NFAT CD16 reporter cells (Fig. 4D), the uninfected HEp-2 cells and HEp-2 cells that were infected with either WT RSV-A or S190R RSV-A were incubated with 7.5 μg/ml nirsevimab for 1 h. Cells were then cocultured with Jurkat NFAT CD16 reporter cells. Measurement of luciferase in the cell supernatant after 6 h of coculture showed that nirsevimab interaction with CD16 led to a significantly higher secretion of luciferase when WT RSV-A-infected cells were used compared to S190R RSV-A-infected cells (Fig. 4E). These data show that the S190R mutation impairs both the neutralization activity and the effector functions of nirsevimab.

### S190R RSV-A shows reduced viral fitness and impaired replication in human organoids

**Figure 5:**
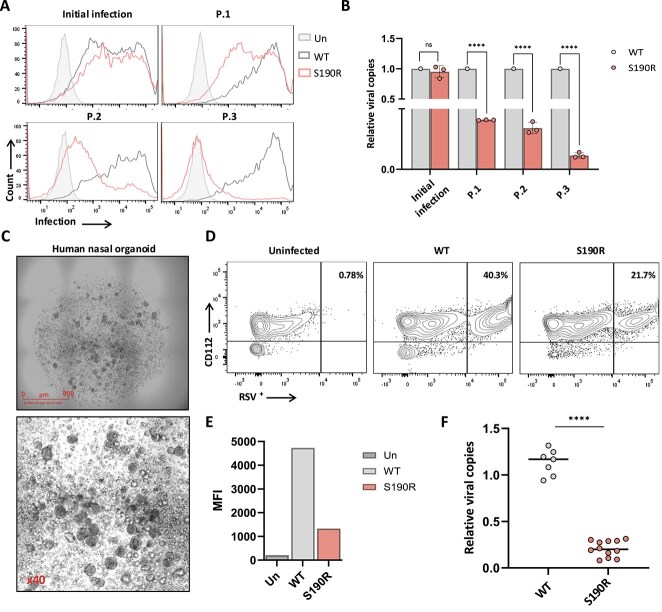
Evaluation of S190R RSV-A fitness. (A, B) Three serial passages of WT RSV-A and S190R RSV-A in HEp-2 cells. The staining of infected cells with RSV3216 is seen in (A). Filled histograms depict the staining of uninfected HEp-2 cells with RSV3216. The viral passage is indicated above each histogram. The relative copy number of RSV-A that was measured by collecting the supernatant in each passage is shown in (B). Data are derived from three biological repeats. Shown are mean values and standard errors. The relative copy number of the WT RSV-A in each passage was set as 1. Statistically significant differences are shown (^*^P < .05, ^***^P < .0005, ns, nonsignificant, Student’s *t*-test). (C) Formation of human nasal organoids that were grown in 6-well plates. Pictures were taken at Day 5 of culture and before RSV infection by using Incucyte® Live-Cell Analysis System. The scale is depicted in the upper panel, and the lower figure is a ×40 magnification of the upper panel. (D) Staining of human nasal organoids with CD112 and RSV3216 72 h after infection with either WT RSV-A or S190R RSV-A. The staining of uninfected organoids is shown in the upper-left histogram. The percentage of CD112^+^RSV^+^ cells is depicted in quadrate Q6. The mean fluorescent index (MFI) of organoids staining with RSV3216 is seen in (E). In (D, E), the figures depict pooled data of three organoids that were separately infected with WT RSV-A or S190R RSV-A. The samples from each group were pooled together before the flow cytometry analysis to ensure sufficient cell numbers. (F) Relative viral load measurements in infected mice. Relative viral load in mice lungs 5 days after infection with WT RSV-A or S190R RSV-A. Each dot depicts a mouse. The *y*-axis indicates fold-change values. Statistically significant differences are shown (^****^P < .0005, ns, nonsignificant, Student’s *t*-test).

Viral escape mutations from neutralizing antibodies frequently result in reduced replication capacity of the mutated virus, as was previously shown for palivizumab escape mutants (Zhao et al. 2006, Lynch et al. 2015, Rosen et al. 2024). This, together with the fact that S190R RSV-A variants were not recorded between 2023 and 2025 in the Global Initiative on Sharing All Influenza Data (GISAID) RSV-A variant databases (Fig. S2) (Shu and McCauley 2017), prompted us to evaluate the viral fitness of S190R RSV-A. To test this, we monitored the replication of WT RSV-A and S190R RSV-A over three passages in HEp-2 cells. For the initial infection, we used adjusted inoculum volumes of WT and S190R virus to ensure equivalent infection and similar genome copy numbers in the cell supernatant and then used equal supernatant volumes for the subsequent sequential infections (passages 1–3). The level of viral replication was evaluated after each passage by staining for RSV-infected cells and by measuring the viral copies in the cell supernatant. S190R RSV-A has demonstrated impaired replication in HEp-2 cells as evidenced by significantly reduced levels of RSV-infected cells across all three viral passages (Fig. 5A), as well as a significantly lower copy number of S190R RSV-A compared to WT RSV-A (Fig. 5B).

To evaluate S190R RSV-A in a more physiologically relevant system, we have isolated nasal epithelial cells from a healthy 35-year-old male and generated primary human nasal organoids (Fig. 5C). After proper formation of the organoids, we have used an equal copy number of WT RSV-A or S190R RSV-A for infection of the organoids. Three days after the infection, the organoids were harvested, and the human epithelial cells (CD112^+^) were evaluated for the percentage of RSV-A-positive cells. We observed 40.3% CD112^+^RSV^+^ positive cells in nasal organoids that were incubated with WT RSV-A, while only 21.7% CD112^+^RSV^+^ positive cells were observed in nasal organoids that were incubated with S190R RSV-A (Fig. 5D and E). Finally, to further support our findings and although RSV-A is a virus adapted to infect and replicate in humans, we infected C57BL/6 mice with WT RSV-A or S190R RSV-A (10^8^ genome copies per mouse). By harvesting the mouse lungs 5 days following infection, we observed a significantly higher viral load in the WT RSV-A–infected mice compared to S190R RSV-A–infected mice (Fig. 5F). Altogether, these results indicate that the nirsevimab escape mutation S190R hinders RSV-A replication.

## Discussion

This study highlights the importance of examining amino acid substitutions across the entire surface of viral proteins following monoclonal antibody administration. Antibody resistance mutations that are removed from the antibody binding site were observed not only in this study but also in previous studies that examined other viral surface proteins (Crowe 2012, Horwitz et al. 2017, Prachanronarong et al. 2019). The HIV-1 envelope (Env) surface protein is the sole target for anti-HIV-1 antibodies and is expressed as a surface trimer of two subunits, the head region (gp120) and the stalk region (gp41). Notably, by introducing a single amino acid change in the Env head region (R456K), HIV-1 can alter the structure of the Env trimer to impair the binding of antibodies that target the stalk region (Horwitz et al. 2017). Moreover, a study by Dound et al. that screened for all influenza virus hemagglutinin mutations that increase the resistance to broadly neutralizing hemagglutinin antibodies indicated that several of the resistance mutations were not located at the antibody binding site but in close proximity to it (Doud et al. 2018). Finally, *in vitro* viral evolution assays of RSV uncovered that a single mutation in the RSV-A F protein, L305I, alters multiple antigenic recognition sites of RSV F antibodies (Oraby et al. 2025).

The isolation of the S190R nirsevimab-resistant variant in this study raises the question of whether this RSV variant will emerge and circulate in the human population due to the selective pressure of nirsevimab. The impaired replication of RSV-A S190R that we observed, together with the fact that this mutation has not been recorded since 2023 in the GISAID databases, argues against this possibility. However, we did demonstrate that the RSV-A S190R is capable of infecting primary human nasal epithelial cells, albeit to a lesser degree than the WT RSV-A. Additionally, it is well documented that viruses, and RSV in particular, are capable of introducing compensatory mutations to counteract the fitness loss caused by an initial mutation (Davis et al. 2009). Thus, we cannot rule out the possibility of the emergence of this or similar nirsevimab-resistant variants.

By analysing antibody binding to epitopes of various RSV-A F antigenic sites, we were able to show that the S190R mutation alters the structure of the RSV-A F trimer. Namely, it hinders antibody access to epitopes located at the antigenic site Ø, as demonstrated by the impaired binding of both nirsevimab and the monoclonal antibody D25. However, the S190R mutation did not affect the other tested antigenic sites of the RSV-A F trimer. The stability and structure of the RSV F trimer have been of great interest due to the fact that the RSV F protein is an extremely unstable viral surface protein in comparison with other viral surface proteins (Battles and McLellan 2019). Moreover, the inability to stabilize the prefusion form of the RSV F trimer was considered to be the main hurdle to generating an effective RSV vaccine (Graham et al. 2015). Of note, the breakthrough discovery demonstrating that two single mutations in the RSV F protein, S190F and V207L, can significantly stabilize the RSV F trimer in its prefusion form, has been instrumental for the development of the RSV vaccines that were approved for use in the elderly population during 2023 (McLellan et al. 2013). Interestingly, the S190R mutation that we have uncovered here is at the same position as the stabilizing mutation S190F. This implies a probable unique importance of the RSV F protein 190 position in the trimer structure and stability. However, our study did not include structural biology techniques such as cryo-electron microscopy (cryo-EM) (Cao et al. 2020), which are essential to accurately determine the structural alteration in the S190R RSV-A F trimers. It will also be intriguing to evaluate the effects of the S190R mutation on the transition of the RSV F trimer from the prefusion state to the postfusion state.

Identification of potential resistant variants is essential for better control of viral outbreaks. Our data indicate that while nirsevimab activity against S190R RSV-A is significantly reduced, palivizumab can effectively bind S190R RSV-A-infected cells. Palivizumab, which targets a different antigenic site of the RSV F protein (antigenic site II), has been used in the clinic since 1998 for RSV prevention in high-risk infants (Moirangthem and Bar-On 2025). As the S190R mutation did not affect palivizumab binding, it supports the possibility of using this antibody against nirsevimab-resistant variants. Alternatively, a combination of several RSV monoclonal antibodies can be used to ensure protection against the variety of RSV strains (Caidi et al. 2012). In this regard, the development of various monoclonal antibodies that recognize different antigenic sites of the RSV F trimer is of great advantage (Widjaja et al. 2016). Since not all of these antibodies are neutralizing antibodies, it is also intriguing to test the possible benefits of giving neutralizing antibodies together with non-neutralizing antibodies that are capable of inducing antibody effector functions and immune-mediated elimination of the infected cells (Bournazos and Ravetch 2017). Of note, passive immunization with a combination of monoclonal antibodies has been proven to improve long-term control of HIV-1, a virus of extremely high diversity compared to RSV, and in which controlling viremia with a single agent is immensely challenging (Bar-On et al. 2018, Mendoza et al. 2018). For example, human studies in HIV-1-infected individuals demonstrated that a combination of two broadly neutralizing antibodies that target distinct viral epitopes can significantly delay viral rebound and even limit the emergence of resistant variants compared to a single broadly neutralizing antibody (Bar-On et al. 2018, Mendoza et al. 2018).

One of the limitations of our study is that the single-genome mutation analysis was done after extended incubation of RSV-A with nirsevimab (starting at Day 12 of culture). Thus, it is possible that the development of the S190R mutation in the RSV F protein requires the prolonged co-evolution of RSV and nirsevimab. Such extended interaction of RSV-A with nirsevimab is not likely to occur during breakthrough infection in infants, despite the long half-life of the antibody (68.7 days) (Hammitt et al. 2022). However, RSV is one of the most common respiratory infections in children, with over 3.6 million hospitalizations annually in children under 5 years of age (Karron 2023). Furthermore, the frequency of breakthrough infection that was recorded in clinical trials and in vaccinated individuals was substantial (Sumsuzzman et al. 2025b). For example, an in-depth study that followed RSV-infected infants during the 2023–24 RSV season reported that out of 695 RSV-infected infants, 346 were administered with nirsevimab (‘Genotypic and phenotypic characterisation of respiratory syncytial virus after nirsevimab breakthrough infections: a large, multicentre, observational, real-world study’, n.d.). Thus, there are considerable numbers of cases of ongoing RSV replication in the presence of nirsevimab in vaccinees. Another caveat of our study is that we did not directly quantify the reduction in nirsevimab binding affinity using biophysical approaches such as SPR (surface plasmon resonance). Performing these assays was not feasible because generating a soluble, stabilized RSV-A F trimer harbouring the S190R mutation proved technically challenging. Future work incorporating these measurements will be essential to precisely define the biophysical impact of this mutation.

The viral evolution experiments we performed do not constitute a comprehensive deep mutational scan, but rather rely on serial passaging of a single viral strain, a strategy that inherently introduces stochasticity into which escape mutations emerge. As a result, certain resistance-associated substitutions, such as mutations at positions previously implicated in nirsevimab escape (Peeples and Thongpan 2023, Xu et al. 2025), may not necessarily arise in our system due to founder effects rather than true biological absence. To mitigate this limitation, we repeated the entire mutation-selection and single-genome sequencing analysis using two independently prepared RSV-A viral stocks, and both experiments consistently identified the S190R mutation as the dominant variant under nirsevimab pressure. Nevertheless, our findings should be interpreted within the context of these constraints, and complementary high-throughput mutational approaches will be important for fully defining the complete spectrum of potential nirsevimab-resistant mutations. A previous study that followed the *in vitro* escape of RSV from nirsevimab indicated a possible escape mutation in the nirsevimab binding site, N67I/N208Y, of the RSV-A F protein (Zhu et al. 2018). In this study, we did not observe an accumulation of such variants, but it is possible that these variants were developed at earlier stages of our assay and were not able to further propagate. Moreover, the lack of nirsevimab-binding site mutants in our assay aligns with the very low frequency of nirsevimab-binding site mutation in RSV-A sequences isolated from two nirsevimab randomized clinical trials (Ahani et al. 2023, Wilkins et al. 2023). This study demonstrated only a single binding site substitution, which was identified in RSV-A-infected vaccines. Moreover, this mutation appeared at a low frequency of 2.1%. Importantly, almost all of the isolated RSV-A viruses had an intact, unmutated nirsevimab binding site (Ahani et al. 2023). In contrast, a relatively higher frequency of around 8% nirsevimab-resistant strains was isolated from individuals who were infected with RSV-B (Fourati n.d., Ahani et al. 2023, Wilkins et al. 2023). This led us to focus our analysis on RSV-A and to identify a novel mutation in the RSV-A F protein that is associated with nirsevimab resistance. However, similar single-genome sequencing analysis should also be performed with RSV-B and with other strains of RSV-A for a more accurate mapping of possible RSV escape mechanisms from nirsevimab. In addition, future studies with more frequent sampling points could provide additional insights into the early dynamics of mutation emergence under nirsevimab pressure.

Finally, the approval of nirsevimab passive immunization for use in healthy individuals marks a unique milestone in controlling viral infection and provides an alternative therapeutic approach against viruses for which an effective vaccine is not available (Moirangthem and Bar-On 2025). The clinical success of nirsevimab could facilitate similar approaches for controlling highly variable RNA viruses such as the influenza virus and HIV-1. Further studies that will be obtained in clinical trials and in preclinical in-depth analysis of the RSV mutation landscape are key for predicting the future success of nirsevimab passive immunization in preventing and controlling RSV infections.

## Supplementary Material

Sup_YB_clean_veag002

## Data Availability

Single-genome sequencing of RSV-A F genes is available and can be accessed under NCBI BioProject PRJNA1365648.
