# Supplementary material for: The S190R mutation in RSV-A F protein impairs nirsevimab binding and neutralization capacity
Source: Virus Evol. 2026 Jan 8;12(1):veag002. doi: 10.1093/ve/veag002 (PMC12878718; doi:10.1093/ve/veag002)
Supplement: Sup_YB_clean_veag002 [file sup_yb_clean_veag002.pdf]

Substitution / Days	RSV-A w/o Nirsevimab			RSV-A + Nirsevimab		
	12	21	39	12	21	39
S190R	0	0	0	1 (0,1)	47 (40,7)	52 (44,8)
N262Y	0	0	0	4 (0,4)	2 (1,1)	0
V76G	2 (0,2)	16 (12,4)	12 (9,3)	2 (0,2)	1 (1,0)	0
S215A	0	0	0	0	0	4 (4,0)
M97T	1 (0,1)	0	0	0	0	0
K327N	1 (0,1)	0	0	0	0	0
E487V	1 (0,1)	3 (2,1)	0	1 (0,1)	0	0
I64T	1 (0,1)	0	0	0	0	0
V533M	1 (0,1)	0	0	0	0	0
I167T	1 (0,1)	0	0	0	0	0
I527V	1 (0,1)	0	0	0	0	0
L45I	0	1 (1,0)	0	0	0	0
K433R	0	1 (1,0)	0	0	0	0
S211G	0	1 (1,0)	0	0	0	0
K123R	0	1 (1,0)	0	0	0	0
K68R	0	1 (1,0)	0	0	0	0
N515Y	0	0	2 (2,0)	0	0	0
N325A	0	0	1 (1,0)	0	0	0
C422Y	0	0	1 (1,0)	0	0	0
D385G	0	0	1 (1,0)	0	0	0
K293R	0	0	1 (1,0)	0	0	0
L512F	0	0	1 (1,0)	0	0	0
S99G	0	0	1 (1,0)	0	0	0
C416R	0	0	0	1 (0,1)	0	0
N165D	0	0	0	1 (0,1)	0	0
Y457F	0	0	0	1 (0,1)	0	0
K124E	0	0	0	0	1 (1,0)	0
I413T	0	0	0	0	1 (1,0)	0
V187A	0	0	0	0	1 (1,0)	0
K75R	0	0	0	0	0	1 (1,0)
WT	36	20	18	40	0	0

Supplemental table 1. A table depicting the number of isolated viral genomes with each mutation. Values in parentheses represent results from two independent experiments, with the number on the left indicating the number of mutant genomes identified in the first experiment and the number on the right indicating those identified in the second experiment.

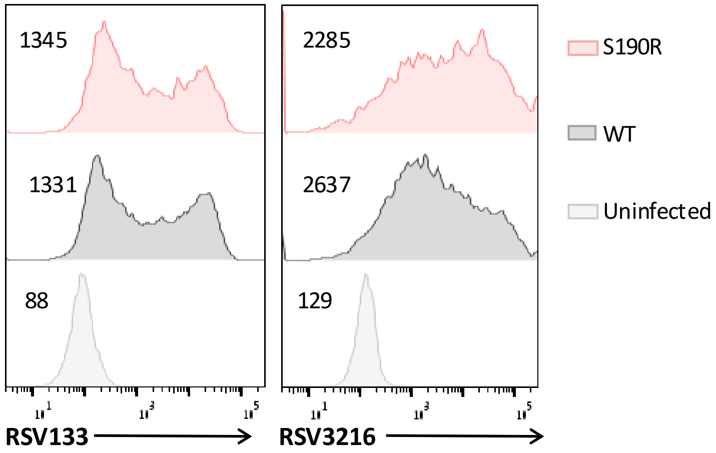

Supplemental Figure 1. Staining of RSV-infected cells with RSV133 and RSV3216 antibodies. Uninfected (light grey), WT RSV-A-infected HEp-2 cells (dark grey), and S190R RSV-A-infected HEp-2 cells (red) stained with either RSV133 (left panel) or with RSV3216 (right panel). The numbers in the figure depict the mean fluorescence index (MFI) that was measured in each histogram. Shown is one experiment out of two independent experiments that were performed. The staining was done 72 hrs after infection with RSV-A.

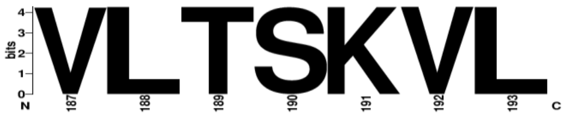

Ref.	VLTSKVL
040-0052	ILTSKVL
040-0011	ILTSKVL
010-0039	ILTSKVL
MN-MDH-RSVA-00532	ILTSKVL
MN-MDH-RSVA-03055	ILTSKVL
010-0011	VLTNKVL
MN-MDH-RSVA-02973	VLTNKVL
PQ535508	VLTSRVL
PQ535507	VLTSRVL

Supplemental Figure 2. A Logo plot and a sequence alignment of RSV-A F protein (position 187-193) from viruses in the GISAID database between January 2023 to May 2025. The frequency of viruses carrying a specific amino acid in the RSV-A F protein is shown by the height of the letter. The numbers in the x-axis depict the amino acid location in the RSV-A2 F protein (UniProt P03420). The figure was made using the web logo tool (https://weblogo.berkeley.edu/logo.cgi). The y-axis height specifies the number of bits, which indicates the information content of a sequence position. By default, the height of the y-axis is the maximum entropy for the given sequence type ($\log_2 20 = 4.3$ bits for protein). The sequence alignment shows the reference sequence and all the variants that were identified, with amino acid substitution marked in red.
